# How does the clinical and tomographic appearance of MRONJ influences its treatment prognosis?

**DOI:** 10.1259/dmfr.20230304

**Published:** 2023-10-23

**Authors:** Catalina Moreno Rabie, Santiago García-Larraín, David Contreras Diez de Medina, Isadora Cabello-Salazar, Rocharles Cavalcante Fontenele, Tim Van den Wyngaert, Reinhilde Jacobs

**Affiliations:** 1 OMFS-IMPATH Research Group, Department of Imaging and Pathology, Faculty of Medicine, University of Leuven, Leuven, Belgium; 2 Department of Oral and Maxillofacial Surgery, University Hospitals Leuven, Leuven, Belgium; 3 Department of Oral and Maxillofacial Radiology, Facultad de Odontología, Universidad de los Andes, Santiago, Chile; 4 Department of Nuclear Medicine, Antwerp University Hospital, Edegem, Belgium; 5 Faculty of Medicine and Health Sciences, University of Antwerp, Antwerp, Belgium; 6 Department of Dental Medicine, Karolinska Institutet, Stockholm, Sweden

**Keywords:** osteonecrosis, cone-beam computed tomography, diagnostic imaging, prognosis, therapy

## Abstract

**Objectives:**

To identify clinical and tomographic prognostic factors for conservative and surgical treatment of medication-related osteonecrosis of the jaws (MRONJ).

**Methods:**

A retrospective search identified patients treated with antiresorptive drugs (ARDs), diagnosed with Stage 1, 2 or 3 MRONJ, and having CBCT scans previous to conservative or surgical treatment. Following data collection, imaging assessment of the following parameters on each MRONJ site was performed: involvement of teeth and/or implants, presence of osteosclerosis, osteolysis, sequestrum formation, periosteal reaction, and pathological fractures. For statistical analysis, patients and lesions were divided into conservative and surgical treatment. Comparisons were made between successful and unsuccessful outcomes. Significance was set at *p* ≤ 0.05.

**Results:**

115 ARD-treated patients who developed 143 osteonecrosis lesions were selected. 40 patients and 58 lesions received conservative treatment, of which 14 patients (35%) and 25 lesions (43%) healed. Additionally, 75 patients and 85 lesions underwent surgery, with 48 patients (64%) and 55 lesions (65%) that healed. Clinical and tomographic risk factors for conservative treatment were MRONJ staging, tooth involvement, extensive osteosclerosis, and deep sequestrum formation (*p* < 0.05). Complementarily, poor prognostic indicators for surgical therapy were a short bisphosphonate (BP) holiday, MRONJ staging, absence of sequestrum formation, and presence of periosteal reaction (*p* < 0.05).

**Conclusions:**

Lesions at Stage 3 MRONJ, with tooth involvement, or sequestrum formation showed poor outcomes when conservative treatment is chosen. Alternatively, surgical treatment is most effective when BPs are discontinued, in Stage 1 lesions, in the presence of sequestrum formation, and absence of periosteal reaction.

## Introduction

Medication-related osteonecrosis of the jaw (MRONJ) can be defined as exposed bone or fistula that probes to bone in the maxillofacial region persisting for more than 8 weeks in patients treated with antiresorptive drugs (ARDs).^1,2^ These medications effectively and safely treat skeletal-related events (SREs) and prevent bone fractures among patients with bone metastases and osteoporosis, respectively.^2–4^ However, whilst diagnostic and treatment methodologies for MRONJ remain debatable, no gold-standard has been agreed upon.^5^

Several efforts have been made to find the best treatment option for MRONJ. As a general thought, the treatment aims to control infection, minimize pain, and avoid necrosis progression.^1,2,6^ Various possible treatment schemes with approaches ranging from conservative to surgical management are indicated based on MRONJ-staging, age, primary disease, comorbidities, and type of ARD. Conservative treatment includes the use of antibiotics and antiseptic mouthwashes but using a variety of compounds and doses.^7^ While the surgical technique mainly consists of removing necrotic and infected bone, softening of the sharp edges, and wound closure with a free-of-tension mucoperiosteal flap. However, some protocols also include the use of laser therapy or local application of autologous platelet concentrates (APC).^7,8^ Therapeutic success is usually considered when reaching mucosal healing in the necrotic site. Yet, success rates have shown different results, being 28.8% in conservative treatment^7^ and ranging from 27.6%^9^ to 91.6%^10^ in surgical removal.

In this context, clinical aspects, including the dosage of ARD, C-reactive protein (CRP), and alkaline phosphate, have been identified as treatment prognostic factors.^5,11^ Nevertheless, few authors have also considered the three-dimensional radiographic appearance of the lesion in this assessment.^5,12–15^ Yet, tomographic images of clinically exposed necrotic bone are variable and may show osteolysis, cortical bone erosion, sequestrum formation, osteosclerosis as well as periosteal reaction.^2,16,17^ In this sense, Shin et al described that osteonecrosis lesions larger than one-third of the jaw had a worse surgical prognosis than smaller lesions.^5^ Likewise, periosteal reaction was also found to be a poor prognostic outcome indicator.^12,13^ However, a better understanding of the factors that predict the post-operative outcome of MRONJ is still necessary.^5,18^

Given the need for a comprehensive assessment using cone-beam CCT (CBCT) to aid treatment prognosis, the present study aims to identify clinical and tomographic prognostic factors for conservative and surgical treatment of MRONJ. A secondary objective is to investigate the imaging features associated with lesion relapse.

## Material and methods

### Study design and settings

Ethical approval was granted by the research ethics committee of University Hospitals Leuven (reference number: S66635). Informed consent was waived given the retrospective longitudinal cohort design. All procedures and data collection were conducted in accordance with the ICH-GCP principles and declaration of Helsinki. The database of the department of oral and maxillofacial surgery at University Hospitals Leuven was reviewed to identify eligible patients between January 1, 2010, and May 31, 2022.

### Participant selection

Patients were included if: (1) older than 18 years, (2) treated with at least one administration of ARDs, (3) diagnosed with Stage 1, 2, or 3 MRONJ according to the American Association of Oral and Maxillofacial Surgeons (AAOMS),^1,2^ and (4) had a CBCT of the MRONJ lesion prior to conservative or surgical treatments. Exclusion criteria included: (1) prior radiotherapy targeted to the jawbones, (2) metastasis in the jaws, (3) Stage 0 MRONJ, (4) absence of documented follow-up (at least two clinical follow-up consultations less than 1 year apart), (5) insufficient image quality to perform their assessment, (6) CBCTs acquired after a surgical procedure, (7) relapse of a preceding MRONJ lesion, and (8) former reconstructive surgery.

### Treatment protocol

All patients were initially given conservative care. Surgical treatment was advised in presence of pain, persistent infection after antibiotic initiation, presence of a mobile sequester, or progression of the lesion’s extension, if the patient’s health status allowed it.

#### Conservative treatment

Conservative treatment involved the prescription of antiseptic mouthwashes, such as 0.12% chlorhexidine or 0.5% sodium hypochlorite during the first 2 weeks of treatment. Subsequently, 0.05% chlorhexidine was used for maintenance throughout the follow-up period. Additionally, amoxicillin 875 mg/clavulanic acid 125 mg or clindamycin 300 mg three times per day were prescribed in the first 2 weeks. Afterwards, the medication was switched to amoxicillin 500 mg or doxycycline 100 mg per day for treatment maintenance until the infection subsided or mucosal healing was achieved. Control visits were initially scheduled every 2 weeks, later transitioning to monthly or 3-monthly appointments, or sooner if the patient experienced worsening symptoms.

#### Surgical treatment

Patients who underwent surgical therapy initially received conservative treatment. Once the outpatient surgery was scheduled, they were prescribed amoxicillin 875 mg/clavulanic acid 125 mg or clindamycin 300 mg three times per day 2 days before surgery. This medication regimen was continued for 2 weeks before transitioning to amoxicillin 500 mg or doxycycline 100 mg per day until the infection subsided or mucosal healing was achieved.

The surgeries occurred under local anesthesia without vasoconstrictor and, in some cases, intravenous sedation (midazolam 0.03 mg/kg and fentanyl 2 μg/kg) was administered. The procedure consisted of wound debridement, sequestrectomy, and occasionally marginal osteotomy of the bone, depending on the extent of the lesion and patients’ symptoms. If teeth or implants were located immediately adjacent to or within the osteonecrosis lesion, they were also removed. For closure, either leukocyte- and platelet-rich fibrin (L-PRF) membranes (408 g/2700 rpm for 12 min; IntraSpinTM, IntraLock^®^, Boca) or a mucoperiosteal free-of-tension flap were placed. The surgical sites were rinsed with 0.9% physiological saline solution and sutured with 3/0 vicryl resorbable sutures. Control visits were scheduled in the same manner as the conservative group.

The primary end point was the presence of mucosal healing during the clinical follow-up. Lesion relapse was noted as a secondary end point. Treatment outcome was assessed in the last documented consultation, and it was considered successful when mucosal healing and absence of symptoms, including swelling, pain, and pus discharge, was achieved. Treatment failure meant a persistent lesion, one that became clinically worse (*i.e.,* stage-up), or an increase in the lesion’s size.

### Data collection

Together with the CBCTs, the following clinical information was collected: age, gender, systemic condition, comorbidities, tobacco and alcohol use, corticosteroid intake, previous chemotherapy and/or radiotherapy, ARD (including dosage and treatment duration), date of MRONJ diagnosis, staging at diagnosis according to the AAOMS,^2^ site of development, oral factors (*e.g.* use of dentures, oral trauma, tooth extraction, etc.), date of CBCT and staging at acquisition, drug holiday (*i.e.* discontinuation of medication at treatment initiation), treatment scheme, surgery date, use of L-PRF, antibiotics, and antiseptic mouthwash, date of mucosal healing, relapse information, and date and staging at follow-up consultations.

### Radiographic assessment

Diagnostic images were acquired at the Dentomaxillofacial Radiology Centre at the Imaging and Pathology Department in St. Raphael Hospital, using 3D Accuitomo 170 (J. Morita Corp., Saitama, Japan) or Newtom VGi evo (Cefla s.c., Imola, Italy). The selection of the field of view (FOV), voxel size (ranging from 80 to 300 µm), and exposure protocol was determined according to the patient’s specific diagnostic or therapeutic indication. Images were assessed using Xero Viewer software (Agfa-Gevaert, Mortsel, Belgium).

A blinded and independent assessment of the CBCT scans was performed by two dentomaxillofacial radiologists and one general dentist. Prior to the commencement of the observations, a calibration session was held using a set of 16 CBCTs involving 22 lesions external to this study to reach baseline diagnostic consensus. All observations were conducted in a quiet room with dim light using a high-resolution display (HP EliteDisplay E243 23.8-inch Monitor; HP inc; Palo Alto). Brightness and contrast setup were left at the discretion of the examiner. In cases where consensus was not achieved, individual discussions were held to reach an agreement. 1 month after completion of the evaluation, 22 CBCTs involving 26 lesions were randomly selected and reassessed to calculate the intraobserver agreement.

The imaging assessment was performed at each MRONJ site. When multiple examinations were available, the CBCT closest to the date of treatment initiation or surgery was selected, depending on whether the patient received conservative or surgical treatment, respectively. The evaluation included the following assessments:

1. Involvement of teeth and/or implants in the lesion, as well as imaging signs of periodontal disease/peri-implantitis. These signs included furcation involvement, horizontal bone loss greater than 1/3 of the root/implant length, angular bone defects, and periapical/peri-implant lesions.^19^ Tooth/implant compromise was considered when immediately adjacent to or embedded in osteolysis, bone sequestrum, or an osteosclerotic area.
2. Osteosclerosis, osteolysis, and sequestrum formation. Considering osteosclerosis as hyperdense areas in the body of the maxilla or mandible; as osteolysis hypodense areas in the cortical and/or trabecular bone; and as sequestrum formation a bony island surrounded by an osteolytic halo. These characteristics were assessed based on depth^14^ and extension.^5^ The lesions were classified as superficial if they were localized to the alveolar process. In contrast, they were considered deep if they extended further than the mandibular canal, maxillary sinus, or nasal cavity. The extension of the lesions was categorized as localized if they were contained in 1/3 of the jaw or generalized if they extended beyond 1/3 of the jaw.
3. Periosteal reaction in the mandible. Considering periosteal reaction as a uniform outer layer of bone formation along the mandibular surface. When present, it was considered localized if it included only the buccal or lingual side of the mandible without involving the lower edge or extensive, if present in both buccal and lingual sides beyond the inferior mandibular border.^20^
4. Pathological fractures.^14^

### Statistical analysis

The collected data were analyzed using RStudio Software v. 2023.3.1.446 (RStudio, Boston, MA). The significance level was set at 5% (*p* ≤ 0.05). Cohen’s (Fleiss) κ test was used to calculate intra- and interobserver agreement. Considering a fair agreement when the test result was ≥0.21–0.40, moderate when ≥0.41–0.60, substantial when ≥0.61–0.80, and almost perfect when ≥0.81–0.99.^21^

Further statistical analysis was conducted to identify clinical and imaging variables that could serve as treatment prognosis predictors. Patients and lesions were initially grouped based on the treatment received, either conservative or surgical. Subsequently, comparisons were made between treatment success and failure. The χ^2^/Fisher’s exact test was used to test the independence of categorical variables, while a Mann–Whitney *U* test was used to assess ordinal variables. The same statistical tests were used to assess the independence of the radiographic features and lesion relapse among both treatment groups.

To examine the relationship between predictor variables and the outcome on each treatment group, a generalized linear mixed model (GLMM) was used. The fixed effects included age, gender, underlying diagnosis, chemotherapy and/or radiotherapy, duration of ARD-therapy, MRONJ staging, presence of teeth and implants, osteosclerosis, osteolysis, sequestrum formation, periosteal reaction, fracture, duration of drug holiday at the start of the treatment, arcade, use of L-PRF, and antibiotics. Patients were included as a random effect to account for multiple lesions per person. A logit link function was used to model the healing probability. LASSO regression was applied to select significant variables, which were then used in a simplified GLMM.

## Results

A total of 115 ARD-treated patients who developed 143 osteonecrosis lesions were included in the present study. They were on average 70 years old (ranging from 43 to 88 years) at the time of diagnosis. Overall, 96 patients (83%) received ARDs in a higher dose for oncologic purposes, while the remaining 19 (17.5%) took lower doses for osteoporosis prevention. Malignancy diagnoses included breast cancer (33.9%, *n* = 39), prostate cancer (22.6%, *n* = 26), multiple myeloma (14.8%, *n* = 17), lung cancer (5.2%, *n* = 6), renal cell cancer (5.2%, *n* = 6), and other types of cancer (1.8%, *n* = 2). Most oncologic patients were treated with both chemotherapy and radiotherapy (53.9%, *n* = 62), whereas others received only chemotherapy (16.5%, *n* = 19), radiotherapy (13%, *n* = 15), or other treatments (16.5%, *n* = 19).

40 patients received conservative therapy and 75 underwent surgery. In the conservative group, 14 patients (35%) healed and required an average of 8.4 months (ranging from 1 to 43 months) from MRONJ diagnosis to mucosal healing, while 26 patients (65%) showed persistence of the lesion and were followed up for an average of 11.5 months (ranging from 1 to 50 months). In the surgical group, 48 patients (64%) healed, which took 14 months (ranging from 1 to 63 months) until achieving mucosal healing. The remaining 27 patients (36%) did not heal during the mean follow-up period of 17 months (ranging from 1 to 61 months). A significantly higher healing rate was observed in patients that received surgical rather than conservative treatment (*p* < 0.05).

Different clinical variables were assessed to explain the treatment outcome. The reason for ARD use, whether it was for osteoporosis or malignancy, did not show a statistically significant difference when comparing treatment outcomes in both the conservative and surgical group (*p* > 0.05). Similarly, age, gender, type-, number-, and duration of ARD, arcade of MRONJ lesion, alcohol consumption, and tobacco use also did not have a significant effect in both treatment groups concerning treatment success (*p* > 0.05). The investigation of the drug holiday at treatment initiation revealed no significant differences in the conservative group, both overall and when analyzing bisphosphonates (BP) and denosumab separately (*p* > 0.05). For the surgical group, no significance was seen in the overall examination nor when isolating denosumab use (*p* > 0.05). Yet, a lengthier BP withdrawal had a significant (*p* < 0.05) effect on the healing outcome, with a mean interruption in healed patients of 21 months and of 2.3 months at patients who had persisting lesions. A summary of these data at a patient level can be found in Table 1.

**Table 1.** Descriptive data of patients with MRONJ receiving conservative and surgical treatment

Characteristic		Conservative treatment					Surgical treatment				
		Healed		Persistent		*p*-value	Healed		Persistent		*p*-value
Number of patients, n (%)		14	35%	26	65%		48	64%	27	36%	
Age at diagnosis (years)	Mean (range)*	64.8 (46– 80)		68.4 (45– 85)		0.347	71.9 (43– 85)		70.5 (50– 88)		0.649
Sex, n (%)	Female	9	41%	13	59%	0.594	31	69%	14	31%	0.404
	Male	5	28%	13	72%		17	57%	13	43%	
Staging at diagnosis, n (%)	Stage 1	7	33%	14	67%	0.164	16	76%	5	24%	0.168
	Stage 2	7	50%	7	50%		30	63%	18	38%	
	Stage 3	0	0%	5	100%		2	33%	4	67%	
Arch, n (%)	Maxilla	5	33%	10	67%	1.000	23	70%	10	30%	0.439
	Mandible	8	36%	14	64%		21	60%	14	40%	
	Both	1	33%	2	67%		4	67%	2	33%	
Time on ARD (months)	Mean (range)*	50.3 (3– 173)		41.8 (10– 119)		0.470	44.7 (1– 240)		32.1 (5– 153)		0.148
Type of ARD, n (%)	Bisphosphonate	4	29%	10	71%	0.698	18	67%	9	33%	0.843
	Denosumab	7	44%	9	56%		23	61%	15	39%	
	Both	3	30%	7	70%		7	70%	3	30%	
Specific ARD, n (%)	Zoledronic Acid	5	25%	15	75%	0.287	17	63%	10	37%	0.292
	Denosumab	10	38%	16	62%		30	63%	18	38%	
	Alendronate	1	25%	3	75%		7	100%	0	0%	
	Pamidronate	1	100%	0	0%		2	67%	1	33%	
	Ibandronate	0	0%	0	0%		1	50%	1	50%	
	Risedronate	1	100%	0	0%		0	0%	0	0%	
Number of ARDs, n (%)*	1	10	36%	18	64%	1.000	39	62%	24	38%	0.394
	2	3	27%	8	73%		9	75%	3	25%	
	3	1	100%	0	0%		0	0%	0	0%	
Drug holiday (months), n (%)	Yes	13	35%	24	65%	1.000	42	62%	26	38%	0.410
	No	1	33%	2	67%		6	86%	1	14%	
	Mean (range)*	5.5 (0–26)		7.2 (0–88)		0.854	10.3 (0–129)		3.4 (0–35)		0.084
Corticosteroid use (months), n (%)	No	10	45%	12	55%	0.230	36	72%	14	28%	0.074
	Yes	4	22%	14	78%		12	48%	13	52%	
	Mean (range)*	26.9 (3– 57)		28 (1–127)		0.956	44.4 (3– 150)		23.4 (1– 78)		0.152
Alcohol consumption, n (%)	No consumption	5	38%	8	62%	0.710	13	62%	8	38%	0.831
	1–2 units weekly	5	45%	6	55%		19	73%	7	27%	
	3–4 units weekly	0	0%	1	100%		1	100%	0	0%	
	≥5 units weekly	1	17%	5	83%		7	70%	3	30%	
	Ex-abuser	0	0%	0	0%		2	50%	2	50%	
	Unknown	3	33%	6	67%		6	46%	7	54%	
Tobacco use, n (%)	Never smoked	4	31%	9	69%	0.815	23	66%	12	34%	0.947
	Active user	4	44%	5	56%		9	64%	5	36%	
	Previous user	4	31%	9	69%		11	61%	7	39%	
	Unknown	2	40%	3	60%		5	63%	3	38%	

ARD, antiresorptive drugs;MRONJ, medication-related osteonecrosis of the jaws.

*p*-values obtained using χ^2^/Fisher’s exact test for categorical data or (*) Mann–Whitney *U* test for ordinal data, to compare at a patient level the treatment outcomes in the surgical and conservative groups. A significant *p*-value was considered when *p* ≤ 0.05.

**Figure 1.**
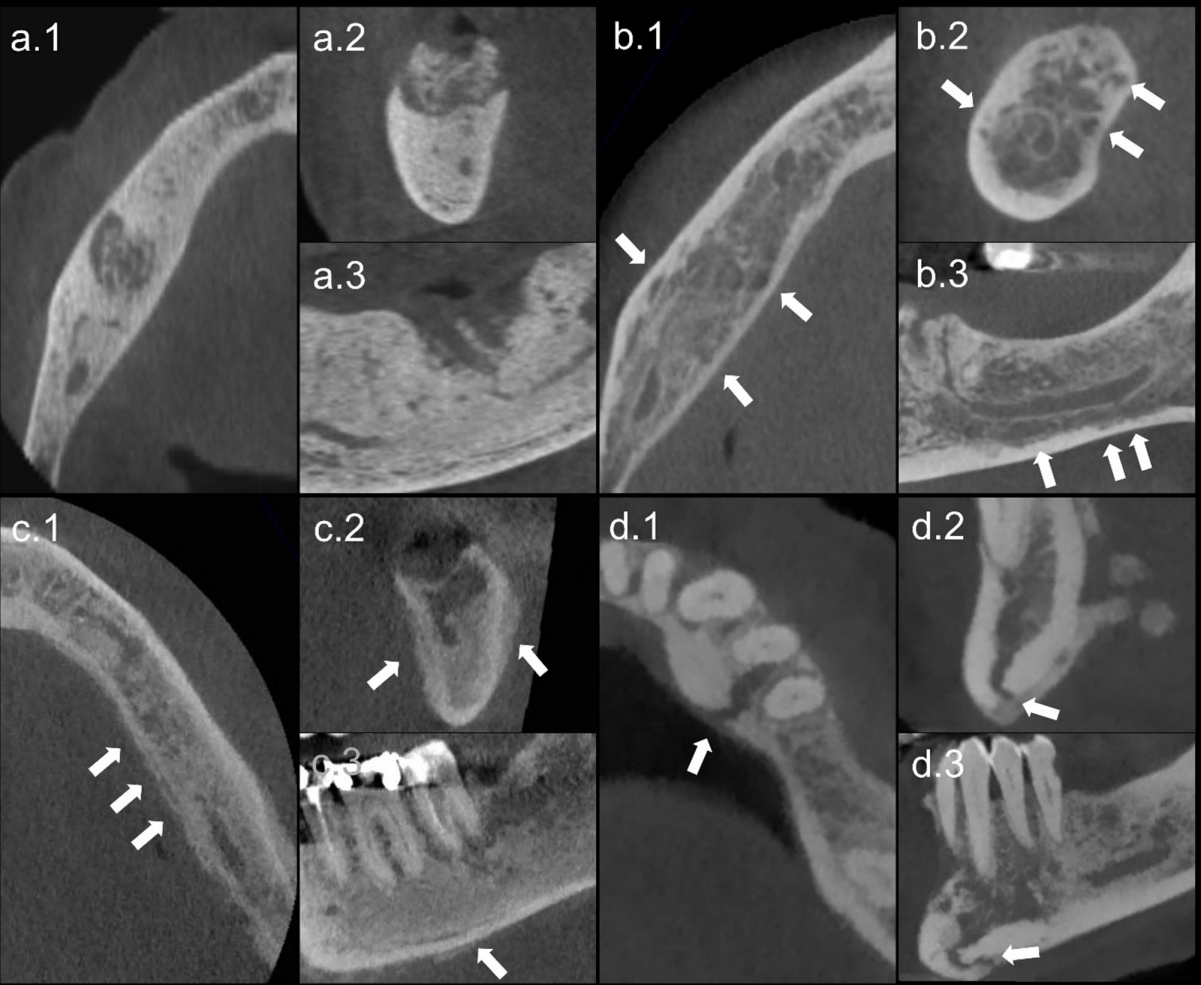
Cropped CBCT reconstructions exampling the imaging features associated with MRONJ in (1) axial, (2) coronal, and (3) sagittal slides of CBCTs. (**a**) Deep and extensive osteosclerosis with a superficial and localized bone sequestrum. (**b**) Deep and extensive osteolytic lesions pointed out with white arrows. (**c**) Extensive periosteal reaction. (**d**) Mandibular fracture. CBCT, cone beam CT; MRONJ, medication-related osteonecrosis of the jaws.

Clinical data regarding lesions in the conservative and surgical group can be found in Table 2 and the results of the CBCT assessment in Table 3. An illustrative example of the observed features can be seen in Figure 1. Overall, intraobserver agreement ranged from substantial to almost perfect (K_OBSERVER 1_=0.828, K_OBSERVER 2_=0.669, K_OBSERVER 3_=0.899) and interobserver agreement was substantial (K_OVERALL_ = 0.725, ranging from 0.691 to 0.744).

**Table 2.** Descriptive data of MRONJ lesions receiving conservative and surgical treatment

Characteristic		Conservative treatment					Surgical treatment				
		Healed		Persistence		*p*-value	Healed		Persistence		*p*-value
Number of sites, n		25	43%	33	57%		55	65%	30	35%	
Staging at treatment initiation, n (%) *	Stage 1	10	42%	14	58%	*0.011*	19	86%	3	14%	*0.028*
	Stage 2	15	58%	11	42%		32	59%	22	41%	
	Stage 3	0	0%	8	100%		4	44%	5	56%	
Arch, n (%)	Maxilla	10	40%	15	60%	0.883	28	74%	10	26%	0.184
	Mandible	15	45%	18	55%		27	57%	20	43%	
Reason for MRONJ	Implant	0	0%	0	0%	0.226	2	67%	1	33%	0.881
	Infected tooth	0	0%	0	0%		1	50%	1	50%	
	Periodontitis	0	0%	0	0%		2	40%	3	60%	
	Prosthesis	4	33%	8	67%		8	73%	3	27%	
	Spontaneous	5	29%	12	71%		10	63%	6	38%	
	Tooth extraction	14	54%	12	46%		29	64%	16	36%	
	NS	2	67%	1	33%		3	100%	0	0%	
Antibiotics, n (%)	Yes	24	42%	33	58%	0.431	54	64%	30	36%	1.000
	No	1	100%	0	0%		1	100%	0	0%	
Use of L-PRF, n (%)	Yes	0	0%	0	0%	NA	36	61%	23	39%	0.409
	No	25	43%	33	57%		19	73%	7	27%	

MRONJ, medication-related osteonecrosis of the jaws; NA, not applicable; ;;NS, Not specified.

The *p*-values described under conservative and surgical treatment correspond to those obtained with the χ^2^/Fisher’s exact test or Mann–Whitney *U* test when data were ordinal (*). Comparisons were made between healed and persistent sites in both treatment groups. Significant *p*-values (*p* ≤ 0.05) are *italicized*.

**Table 3.** Three-dimensional imaging assessment of MRONJ lesions that received conservative and surgical treatment

Characteristic		Conservative treatment					Surgical treatment				
		Healed		Persistence		*p*-value	Healed		Persistence		*p*-value
Number of sites, n		25	43%	33	57%		55	65%	30	35%	
Teeth	Absent	23	52%	21	48%	*0.029*	41	63%	24	37%	0.765
	Present	2	14%	12	86%		14	70%	6	30%	
Implants	Absent	25	44%	32	56%	1.000	51	64%	29	36%	0.652
	Present	0	0%	1	100%		4	80%	1	20%	
Osteosclerosis depth*	Absent	10	59%	7	41%	0.147	10	67%	5	33%	0.779
	Superficial osteosclerosis	2	40%	3	60%		2	40%	3	60%	
	Deep osteosclerosis	13	36%	23	64%		43	66%	22	34%	
Osteosclerosis extension*	Absent	10	59%	7	41%	*0.010*	10	67%	5	33%	0.906
	Localized osteosclerosis	11	55%	9	45%		22	65%	12	35%	
	Extended osteosclerosis	4	19%	17	81%		23	64%	13	36%	
Osteolysis depth*	Absent	12	44%	15	56%	0.959	18	55%	15	45%	0.249
	Superficial osteolysis	5	38%	8	62%		14	74%	5	26%	
	Deep osteolysis	8	44%	10	56%		23	70%	10	30%	
Osteolysis extension*	Absent	12	44%	15	56%	0.663	18	55%	15	45%	0.210
	Localized osteolysis	12	46%	14	54%		32	73%	12	27%	
	Extended osteolysis	1	20%	4	80%		5	63%	3	38%	
Sequester depth*	Absent	13	57%	10	43%	*0.036*	13	50%	13	50%	*0.020*
	Superficial sequester	11	42%	15	58%		26	65%	14	35%	
	Deep sequester	1	11%	8	89%		16	84%	3	16%	
Sequester extension*	Absent	13	57%	10	43%	0.090	13	50%	13	50%	0.063
	Localized sequester	12	36%	21	64%		39	70%	17	30%	
	Extended sequester	0	0%	2	100%		3	100%	0	0%	
Periosteal reaction*	Absent	22	46%	26	54%	0.392	52	70%	22	30%	*0.008*
	Localized reaction	2	40%	3	60%		0	0%	2	100%	
	Extended reaction	1	20%	4	80%		3	33%	6	67%	
Fracture	Absent	25	45%	31	55%	0.501	55	65%	30	35%	NA
	Present	0	0%	2	100%		0	0%	0	0%	

MRONJ, medication-related osteonecrosis of the jaws.

*p*-values obtained using χ^2^/Fisher’s exact test for categorical data or (*) Mann–Whitney *U* test for ordinal data when comparing healed and persistent sites in the conservative and surgical group. Significant values are marked in *italic* (*p* ≤ 0.05).

### Conservative treatment

From a total of 143 lesions, 58 (41%) received conservative treatment, and 25 (43%) of them achieved healing. Among the 58 lesions, 10 (17%) experienced recurrence, and out of those, only three remained unhealed. MRONJ lesions that received conservative treatment were monitored for a mean of 16 months (ranging from 1 to 59 months). Healing occurred on average 10 months (ranging from 1 to 36 months) from the start of treatment until mucosal healing was first observed. CBCT scans were acquired on average 2.6 months after diagnosis (ranging from 0 to 16 months). When evaluating the clinical risk factors, only the staging of the lesion showed significant results. Lesions in Stage 1 and 2 showed a significant healing (circa 50%) compared to absence of healing seen in Stage 3 lesions (*p* < 0.05).

**Figure 2.**
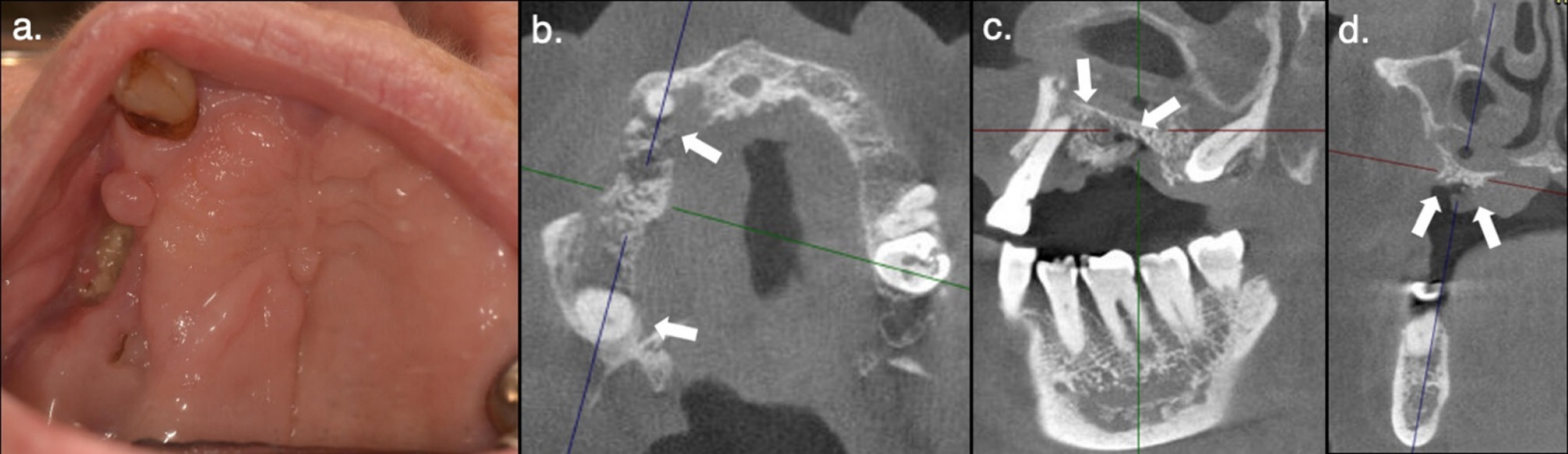
Conservative treatment of osteonecrosis. An 84-year-old female with breast cancer and bone metastases, under denosumab treatment, presented with a year-old osteonecrosis lesion (**a**). Clinically, there was increased mobility in the upper right canine, absence of pain, oronasal or antral communication, inflammation, or suppuration. Conservative treatment was initiated and a CBCT image was taken. Axial (**b**), sagittal (**c**), and coronal (**d**) CBCT slices revealed tooth involvement (b, white arrows), localized osteosclerosis, and bone sequestrum involving the lower nasal wall (c, d, white arrows). At 6-month follow-up, loosening of bone fragments was reported, but the lesion showed minor changes. Subsequent visits were not attended due to the patient’s deteriorating health.

Tomographic characteristics that indicated resistance to treatment included lesions with tooth involvement as 86% of these lesions did not heal, hyperdense trabecular pattern extending to more than 1/3 of the mandible or maxilla with 81% of these lesions persisting, and presence of sequesters involving the maxillary sinus or mandibular canal as 89% of these lesions remained unhealed during follow-up (*p* < 0.05) (Figure 2). It is worth noting that from the 14 teeth involved in lesions, 9 had periapical radiolucency or radiographic signs of periodontal disease. Moreover, none of the studied imaging features showed an association with lesion relapse (*p* > 0.05).

The variables selected for the GLMM were age, teeth involvement, osteosclerosis extension, and sequester depth. Results indicate that older age, tooth involvement, an extensive osteosclerosis, and deep sequesters significantly decrease the chance of achieving mucosal healing after conservative treatment (*p* < 0.05).

### Surgical treatment

A total of 85 lesions (59%) received surgical treatment resulting in 55 lesions (65%) that healed. Among these 85 lesions, 9 (11%) had relapse after surgery, with 2 of them remaining unhealed. The surgeries were performed on average 7 months (ranging from 0 to 64 months) after the initiation of conservative treatment, and 61 (72%) of lesions underwent surgical procedure within 6 months of conservative treatment initiation. The mean follow-up duration for this treatment group was of 25 months (ranging from 1 to 106 months) and mucosal healing was first achieved in average 16 months (ranging from 1 to 83 months) after diagnosis and 7 months (ranging from 0.4 to 40 months) after surgery. CBCT examinations took place in an average of 2.3 months (ranging from 0 to 10 months) prior to surgery. In terms of lesion staging, a significant association was found, as lesions in Stage 1 achieved the highest healing rate after surgery (86%), with rates diminishing in Stage 2 (59%), and 3 (44%) (*p* = 0.028).

**Figure 3.**
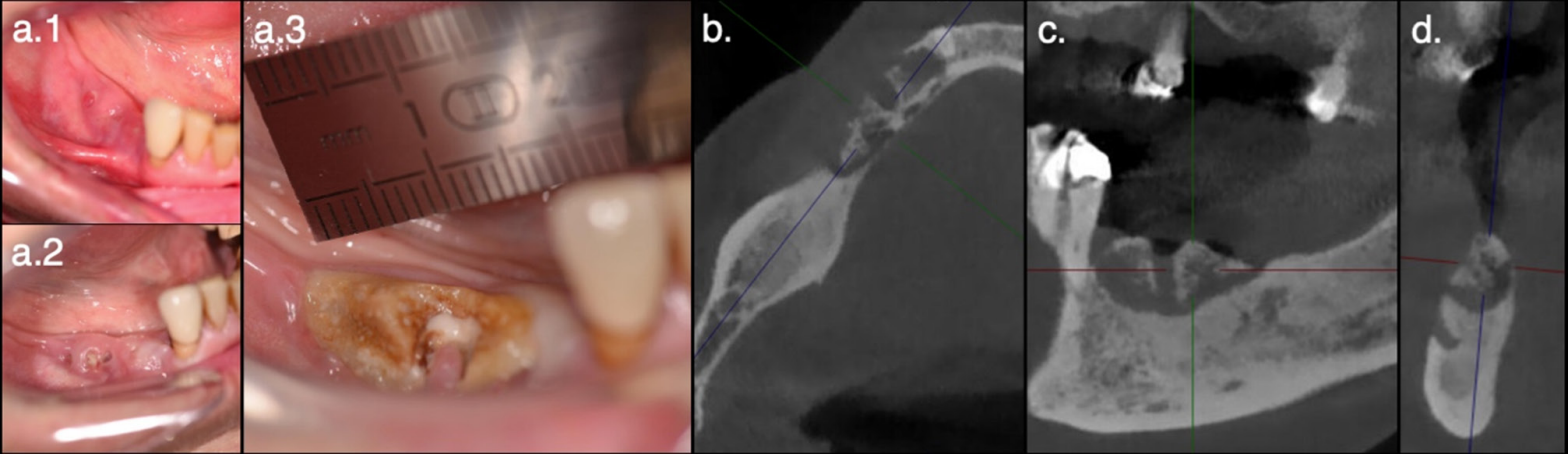
Surgical management of osteonecrosis. A 70-year-old female with breast cancer and bone metastases, treated with denosumab, presented with MRONJ following tooth extractions of the lower right premolars. Initially, there was a small fistula probing to bone accompanied by pain and suppuration (a.1). Despite antibiotic and antiseptic mouthwash administration, the lesion progressed (a.2, 1 month and, a.3, 3 months after diagnosis). Axial (**b**.), sagittal (**c**.), and coronal (**d**.) CBCT slices show involvement of the lower right canine, deep and extensive osteosclerosis, and superficial and localized sequestrum formation. Sequestrectomy and L-PRF application under sedation were performed 1 month after CBCT acquisition (clinical photograph a.3.). Mucosal healing and symptom resolution was observed 3 months later. No relapse was documented. MRONJ, medication-related osteonecrosis of the jaws.

Tomographic characteristics associated with persistence of the lesion after treatment were the absence of sequestrum formation and the presence of periosteal reaction, while lesions with sequestrum formation presented the most success (*p* < 0.05) (Figure 3). Additionally, no imaging feature under study revealed an association with lesion relapse (*p* > 0.05).

The selected variables for the GLMM were gender, duration of ARD treatment, MRONJ staging at the start of treatment, presence of implants, osteolysis extension, sequestrum depth, periosteal reaction, drug holiday, arcade, and use of L-PRF. In the tailored model, only a worse MRONJ staging showed a significant association with a poorer treatment outcome (*p* < 0.05).

## Discussion

The present study aimed to identify clinical and three-dimensional imaging findings that serve as therapeutic prognosis risk factors for MRONJ. Our sample included 58 lesions treated non-operatively and 85 lesions treated surgically. Risk factors found for conservative treatment were MRONJ staging, presence of teeth in the lesion, extensive osteosclerosis, and deep sequestrum formation. Complementarily, poor prognostic indicators for surgical therapy were MRONJ staging, length of BP holiday, absence of sequestrum formation, and presence of periosteal reaction.

The choice of whether to recommend conservative or surgical treatment is still a matter of debate.^13^ The AAOMS suggests that both approaches can be considered for all clinical stages judging on the disease progression and patient’s comorbidities.^2^ Similar recommendations are given by the Japanese position paper.^22^ Even though, systematic reviews have indicated that surgical treatment offer superior results to those of conservative therapy.^18,23,24^ For this reason, Kawaoka et al recommend surgical therapy as first choice in all MRONJ stages.^13^ In the current investigation, the success rates for non-operative and operative treatment were found to be 43 and 65%, respectively. These rates are comparable to those reported in prior publications ranging from 25 to 46% in conservative treatment^7,11,18,25,26^ and from 28 to 92% in surgical therapy.^5,9–11,23,27^

Conservative treatment for MRONJ is proposed to provide symptom relief rather than to reach complete mucosal healing because necrotic bone is unlikely to heal spontaneously. Yet, this approach implies a long-term management, which can potentially lead to progression of the pathology.^11^ Although, a Canadian study described that patients’ quality of life improved with conservative treatment even if it did not resolve the pathology due to symptom relief.^28^ Our results show that despite having asymptomatic patients with unchanged lesions, mucosal healing is improbable if teeth and sequester formation are involved. Moreover, those teeth are often affected by endodontic and/or periodontal disease, which in turn are risk factors for the onset of MRONJ.^29,30^ Therefore, tooth extraction is advisable in these patients.

Surgical therapy for MRONJ has been associated with successful outcomes and significantly less recurrence than non-surgical therapy.^27^ Variables that could compromise the prognosis are diabetes,^13^ extensive osteolysis,^5,27^ absence of sequester,^12^ severe osteosclerosis,^12,13^ presence of periosteal reaction,^12,13^ absence of drug holiday,^11,12^ and a history of high-dose antiresorptive therapy with either bisphosphonates or denosumab.^11–13^ All these findings are consistent with our results. Additionally, a systematic review described higher healing rates in Stages 1 and 2 (72% and 79%, respectively) than in Stage 3 (27%) with less invasive surgical approaches,^23^ which is also supported by our findings.

Once the surgical approach is chosen to treat MRONJ, the extent of the resection and use of healing aids like L-PRF or hyperbaric oxygen are at discretion of the surgeon. It has been reported that a better prognosis is associated with extensive surgical removal in contrast to a minimally invasive procedure.^5,11,18,23^ In our hospital, a less invasive surgical approach is often opted, which may explain the moderate success rates. Besides, when sequesters are absent, determining the appropriate resection size becomes challenging. In such instances, radiographic identification of osteosclerosis can serve as a helpful guide.^15^ Particularly, when observing periosteal reaction, Kawaoka et al suggested to remove its complete extent as healing was reached in 83 and 61% of the cases with complete and partial resection, respectively.^13^ In parallel, Kojima et al investigated the factors related to periosteal reaction and found that mandibular osteonecrosis, severe osteosclerosis, and a diagnosis of malignancy were significantly related to this feature.^12^

The absence of a clinicoradiographic evaluation in the AAOMS categorization of osteonecrosis has drawn criticism.^31^ Given that imaging features such as osteolysis, cortical bone erosion, sequestrum formation, and osteosclerosis can occur at any clinical Stage,^2^ and this was also seen in our sample, which challenges the clinical staging-based treatment decision. For instance, a lesion that clinically displays no evidence of infection or inflammation may show imaging signs of these, such as periosteal reaction, osteolysis, or a radiopaque maxillary sinus. In addition, MRONJ lesions and their imaging findings may change over time influencing the treatment’s prognosis, which makes imaging diagnostic tools vital to consider.^15^

Limitations of this study include those related to its retrospective nature, such as heterogeneity in the data due to surgical variability, differences in the drug scheme, and comorbidities of the included patients. Furthermore, the data belongs to only one treating center, thus providing a restricted sample. Nevertheless, despite these limitations, this study provides evidence that favors operative treatment when encountering lesions in Stage 3, with teeth, or sequesters involved. Likewise, surgical treatment showed outstanding results in Stage 1, but significantly reduced its effectiveness in Stages 2 and 3. Finally, if the latter is chosen and the patient’s health status allows it, a drug holiday, especially under BP use, seems beneficial to the outcome. Further investigations should be carried out to confirm the present findings and assess suitable treatment alternatives for MRONJ lesions showing resistance to surgical treatment, which presented absence of sequestrum or presence of periosteal reaction on CBCT.

## Conclusion

To conclude, this study reports a comprehensive assessment of risk factors for conservative and surgical management of MRONJ. Conservative treatment yielded poor outcomes for lesions at Stage 3 MRONJ, with tooth involvement, or sequestrum formation. Conversely, surgical treatment demonstrated its highest effectiveness for Stage one lesions, particularly when bisphosphonates were discontinued, and in cases with sequestrum formation, and absence of periosteal reaction.
